# Greater nitrogen dioxide concentrations at child versus adult breathing heights close to urban main road kerbside

**DOI:** 10.1007/s11869-015-0370-3

**Published:** 2015-09-15

**Authors:** Hannah S. Kenagy, Chun Lin, Hao Wu, Mathew R. Heal

**Affiliations:** School of Chemistry, University of Edinburgh, David Brewster Road, Edinburgh, EH9 3FJ UK

**Keywords:** Nitrogen dioxide, Exposure, Air pollution, Child, Passive sampler, Urban traffic

## Abstract

Nitrogen dioxide (NO_2_) is a ubiquitous air pollutant with high concentrations particularly close to main roads. The focus of this study was on possible differences in NO_2_ concentrations between adult and child heights as a function of different distances from heavily trafficked roads in urban areas. Passive diffusion tubes were used to measure NO_2_ concentrations at heights of 0.8 m (approximate inhalation height of children and closer to vehicle exhaust height) and 2.0 m (approximate inhalation height of adults) above the ground at a number of locations and over several weeks in the city of Edinburgh, UK. Evidence for significant differences in NO_2_ between heights was observed up to at least 1.2 m from kerbside of busy roads, with tubes at 0.8 m measuring concentrations 5–15 % (a few μg m^−3^) greater than at 2.0 m. The vertical NO_2_ concentration difference was not observable at distances 2.5 m or greater from the kerbside. Fitting of horizontal transects of NO_2_ concentrations away from main roads demonstrated the strong influence of wind speed in yielding faster fall-off in NO_2_ concentration from the roadside, and in near-ground vertical gradient in NO_2_, and lower background NO_2_ concentrations. These observations have potential public health implications for differential NO_2_ exposures between children walking, or in buggies, close to heavily trafficked urban roads compared with adults.

## Introduction

Nitrogen dioxide (NO_2_) is a ubiquitous air pollutant that has both adverse short- and long-term effects on human health (WHO 2006, 2013). Short-term variations in NO_2_ have been linked to increased hospitalisations, respiratory symptoms, and mortality, while long-term exposure to NO_2_ has also been associated with both morbidity and mortality (WHO 2006, 2013). In urban areas, where the majority of population exposure to ambient NO_2_ occurs, the major source of NO_2_ is traffic exhaust, both through directly emitted primary NO_2_ and through the fast reaction between emitted NO and ambient ozone (O_3_). The latter reaction happens on comparable timescales to dispersion away from the road source. Consequently, considerable horizontal spatial variation in NO_2_ in urban areas, associated with distance from roads, has been reported (e.g. Vardoulakis et al. 2009; Cyrys et al. 2012; Caballero et al. 2012; Dedele and Miskinyte 2015).

In contrast, near-ground vertical variation in NO_2_ has received very little attention. Exhaust pipes on motor vehicles are generally located fairly close to the ground. Depending on how fast mixing occurs, this could have important implications on the exposures to NO_2_ (and to other traffic-related pollutants) of children versus adults along streets. The breathing height of children, when either walking or riding in a buggy (stroller/pushchair), is much lower than the average adult breathing height. Typically, pollution measurements are made at or just above adult breathing height and therefore do not account for the possibility of a difference in exposure for children and adults. Additionally, it is suggested that the developing bodies of children are more susceptible to the adverse health effects of air pollution than are adults (WHO 2005), and exposure to a greater concentration could compound this problem. A recent meta-analysis has quantified the impact of traffic-related pollution, as characterised by NO_2_, on asthma prevalence in children (Favarato et al. 2014).

Thus, the aim of this study was to investigate NO_2_ concentrations at heights relevant to adult and child inhalation close to, and on horizontal transects away from, urban main roads. As far as we are aware, this is the first such investigation of near-ground vertical differences in NO_2_ concentrations. Laxen and Noordally (1987) and Monn et al. (1997) investigated vertical profiles of NO_2_ in a street canyon, but the lowest measurement heights in each case were 2.6 and 3.5 m, respectively. In a larger study of horizontal variation of air pollutants in Paris, Vardoulakis et al. (2005) performed one set of measurements of BTX pollutants at 1.5 m and higher heights on both sides of a street canyon in Paris. In a study with a similar objective to this one, Galea et al. (2014) concluded that PM_2.5_ concentrations measured with a portable monitor at a child-in-a-buggy breathing height were not significantly greater than concentrations measured concurrently with a second monitor at an adult height.

In this study, duplicates of passive diffusion tubes (PDTs) were used to measure concentrations of NO_2_ simultaneously at 2.0- and 0.8-m heights at a number of locations over several weeks in the city of Edinburgh, UK.

## Methods

Palmes-type PDTs were deployed in four sampling designs (described below) between mid-July and mid-November 2014 in Edinburgh, a city of ∼490,000 population situated near the east coast of Scotland, UK (55.9° N, 3.2° W). The different sampling designs covered a range of near-roadside locations and transects away from a major road, and each design was replicated two to five times. At every location, duplicate tubes were placed at both 0.8 and 2.0 m from the ground, approximately representative of child and adult inhalation heights, respectively. The latter is slightly higher than the adult breathing height of ∼1.7 m but is the height recommended in UK national guidance to local authorities for measurement of NO_2_ by a passive diffusion tube (Defra 2009) (to limit theft), so measurements in our study at this height are consistent with measurements made elsewhere in the UK for local air quality assessment.

All tubes were exposed for 1 week. The preparation and analysis of all PDTs followed UK national protocol (Defra 2008). PDT components were obtained from Gradko International Ltd. (www.gradko.com), adsorbent meshes were prepared by soaking in 50 % v/v triethanolamine/acetone solution, and accumulated nitrite in exposed tubes was determined via the Saltzman reaction and optical absorbance at 540 nm.

**Fig. 1:**
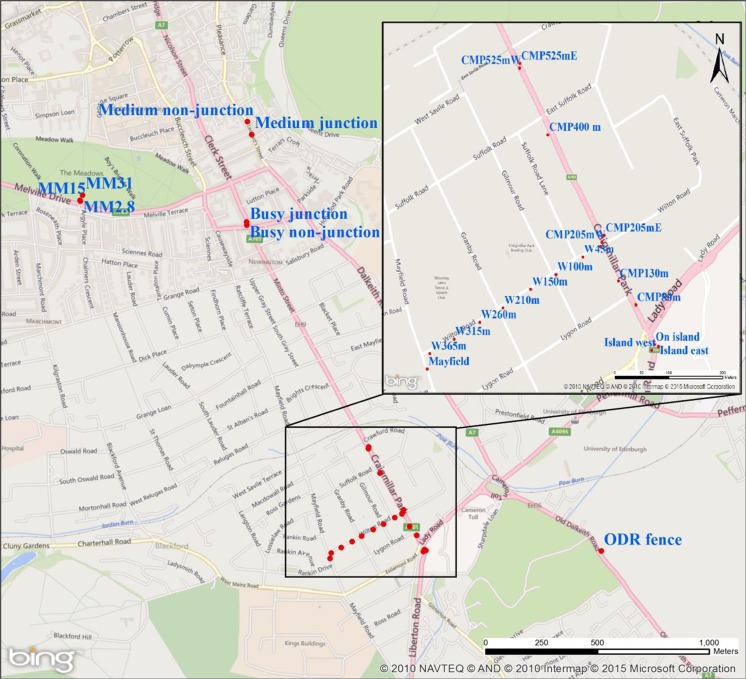
Map of south-central Edinburgh with locations of the dual-height NO_2_ measurements. The centre of the city is top of the map. Wind speed was measured at King’s Buildings, towards the bottom of the map. Map source as given in the figure

A map of the PDT sampling locations in south-central Edinburgh is shown in Fig. 1. For two exposure weeks, PDTs were placed on a small transect along a walking path (Middle Meadow Walk) into a park (The Meadows) at 2.8-, 15-, and 31-m horizontal distances (sites MM2.8, MM15, and MM31) from a busy road (Melville Drive, annual average daily vehicles (AADV) 13,695 in 2014; traffic data from www.dft.gov.uk/traffic-counts). Additionally, two locations along a busy road (South Clerk St, AADV 12,742 in 2014), at a junction and away from a junction (sites Busy junction and Busy non-junction), and two locations along a medium-busy road (St. Leonard’s Street), at a junction and away from a junction (sites Medium junction and Medium non-junction), were selected. These sites were located between 0.6 and 0.9 m from the kerbside.

For four exposure weeks, PDTs were located along the heavily trafficked Craigmiller Park Road (AADV 12,844 in 2014). Three sites were at the junction with Lady Road, including on a traffic island in the intersection (site On Island) and on either side of Craigmiller Park Road opposite the traffic island (sites Island West and Island East); two sites were 205 m north of this intersection on either side of Craigmiller Park Road (sites CMP205mW and CMP205mE); and two sites were 525 m north of the intersection (sites CMP525mW and CMP525mE). These sites were in the range 0.4–1.2 m from the kerbside.

For five exposure weeks, PDTs continued to be placed at the sites along the west side of Craigmiller Park Road with additional tubes located at 80, 130, and 400 m north of the junction with Lady Road (sites CMP80m, CMP130m, and CMP400m). PDTs were also placed at 4.7 m and then every 40–60 m on a full transect along Wilton Road, a minor residential road with little traffic extending perpendicularly west from Craigmiller Park Road to its junction with another main road, Mayfield Road (sites W4.7m, W45m, W100m, W150m, W210m, W260m, W315m, W365m, and Mayfield).

For two exposure weeks, deployment was in the form of a high-resolution horizontal transect at the two heights along a fence bordering a park extending perpendicularly from another busy road, Old Dalkeith Road (AADV 13,679 in 2014) (location ODR fence on Fig. 1). The first site was at 2.5 m from the kerbside, and thereafter, tubes were located at up to 0.5-m horizontal resolution up to 6.7 m from the kerbside and then at coarser horizontal resolution up to a few tens of metres from the kerbside. A site directly across Old Dalkeith Road opposite the perpendicular transect provided an additional measurement site 1.1 from the kerbside of this road.

All PDT values were used with no selection criteria applied. Occasionally, deployed tubes were stolen or vandalised, or data were otherwise missing. Both replicate data were missing for only 8 deployments. For 13 deployments, one duplicate value was missing and the value of the remaining tube was used directly. In all other cases, the mean of the duplicates was used. The final dataset comprised 142 pairs of NO_2_ concentrations at the two heights above the ground, of which 84 pairs were located within 5 m of the kerbside.

Evidence for differences in NO_2_ concentration between the two heights was evaluated via a paired *t* test. The rate of horizontal NO_2_ concentration fall-off from the kerbside was investigated through the fitting (using Matlab) of a three-parameter exponential decay model to concentrations along a horizontal transect.

## Results and discussion

The mean (±1 SD) relative standard deviation between duplicate tubes for all measurements made during this study was 6.5 ± 2.1 %. This is well within the acceptable RSD range typically reported in the literature (Cape 2009).

A paired *t* test on measurements at 0.8 and 2.0 m above the ground for all PDT deployments less than 5 m from the kerbside (a working definition of ‘roadside’ for local air quality assessment (Defra 2009)) demonstrated highly significant evidence for a difference in NO_2_ concentration between the two heights (*p* = 8 × 10^−12^, *n* = 84, Table 1). Concentrations at 0.8 m above the ground were, on average, 5.2 μg m^−3^ greater than at 2.0 m above the ground (average concentrations at the two heights were 57.2 and 52.0 μg m^−3^, respectively). The average relative difference in concentration (difference divided by mean) was 8.7 %. In contrast, a paired *t* test on measurements at the two heights at locations greater than 5 m from the kerbside showed no significant evidence for a difference in NO_2_ concentration between the two heights (*p* = 0.08, *n* = 58, Table 1). For these data, the average difference in NO_2_ concentration between 0.8 and 2.0 m was 0.8 μg m^−3^ (or 2.0 %), in the context of average concentrations of 22.8 and 23.6 μg m^−3^, respectively.

**Table 1:** Summary of differences in NO_2_ concentrations between 2.0 and 0.8 m above the ground at the same location. The dataset is divided into a number of sub-groupings based on distance from the kerbside. The results from paired *t* tests (two-tailed) on the data are also shown. Statements on significance are with respect to the 95 % confidence level

	Number	Average (±SD) difference/μg m^−3^	Average (±SD) difference/%	*p* value	Significant?
All data <5 m from kerb	84	−5.2 (±6.0)	−8.7 (±11.9)	7.9 × 10^−12^	Yes
All data >5 m from kerb	58	0.77 (±3.24)	2.0 (±14.0)	0.077	No
0.4 m from kerb	13	−7.94 (±5.23)	−14.4 (±9.5)	1.4 × 10^−4^	Yes
0.5 m from kerb	19	−7.46 (±3.89)	−14.8 (±6.7)	1.3 × 10^−7^	Yes
0.6 m from kerb	8	−3.54 (±4.34)	−5.3 (±5.7)	0.055	No
0.7–0.9 m from kerb	10	−3.31 (±4.41)	−6.6 (±8.0)	0.042	Yes
1.0–1.1 m from kerb	11	−5.16 (±5.54)	−9.8 (±10.6)	0.012	Yes
1.2 m from kerb	9	−10.0 (±7.16)	−11.6 (±8.9)	0.003	Yes
2.5–3.5 m from kerb	4	1.32 (±5.08)	6.2 (±16.4)	0.639	No
3.8–4.7 m from kerb	10	1.03 (±5.44)	3.7 (±17.2)	0.562	No

Since the above analysis is dominated by measurements closer than 5 m to the kerbside, the dataset was further categorised by distance from the kerbside in order to determine the distance from the kerbside at which there was no evidence of significant difference in NO_2_ concentration with height above the ground (Table 1). Paired *t* tests on the data showed that a significant difference in NO_2_ concentration with height persisted for all categories through 1.2 m from the kerbside (with the exception of 0.6 m from the kerbside where *p* = 0.055), with average relative differences of 5–15 %. For the category of 2.5–3.8-m distance from the kerbside, and greater distances, the paired *t* test no longer showed evidence for a difference in NO_2_ concentrations between the two heights. These observations clearly have potential public health implications as they show that children walking, or in buggies, close to busy roads can be exposed to NO_2_ concentrations several percent (or a few μg m^−3^) greater than adults at the same locations. However, it is acknowledged that the use of 2.0 m as the upper height in this study, being slightly higher than the adult breathing zone, may be over-emphasising differences in NO_2_ concentration between child and adult breathing heights near the roadside. It is also acknowledged that adults are likely to try and keep small children and buggies away from the immediate kerbside where possible. Also, concentration of pollutant alone does not define proneness to developing adverse health effects; for example, breathing rate and susceptibility are also important (but if children are more sensitive to air pollution, then the observation of greater NO_2_ concentrations at lower heights close to busy roads is relevant).

**Fig. 2:**
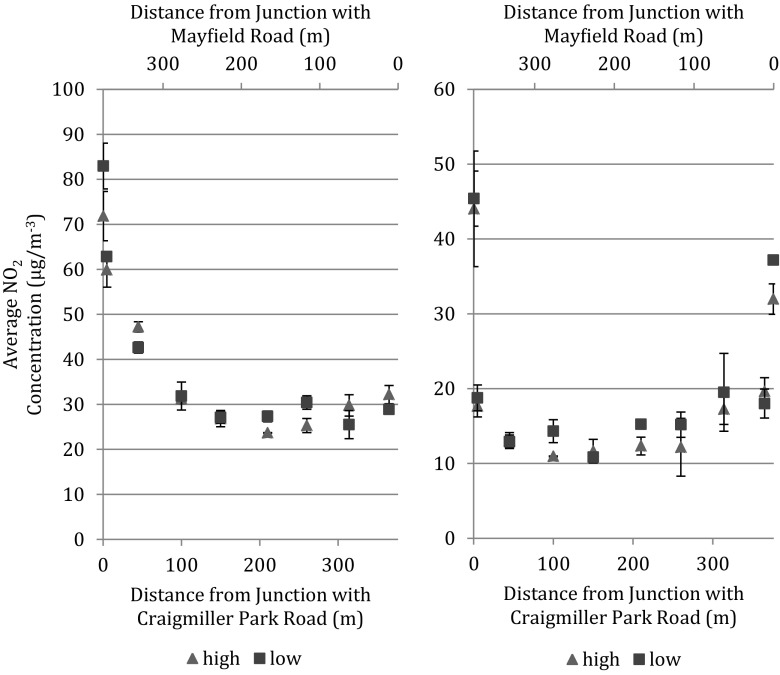
Concentrations of NO_2_ along Wilton Road between Craigmiller Park Road and Mayfield Road in weeks with average wind speeds of (*left*) 0.76 m s^−1^ and (*right*) 3.27 m s^−1^. *Uncertainty bars* are ±1 SD of duplicate tubes. Unfortunately, data at the Mayfield Road end are missing in the *left-hand figure*

The high horizontal resolution dual-height NO_2_ measurements taken along a footpath perpendicular to Old Dalkeith Road showed a significant vertical concentration differential to 1.1 m from the kerbside in one set of exposures and to 2.5 m in the second set. This difference reflects the difference in average horizontal wind speed during the exposures (1.91 and 0.79 m s^−1^, respectively), affecting dispersion from the vehicle exhausts, which is investigated in more detail using data from the transects along Wilton Road. (Wind data were obtained from a met station at the King’s Buildings campus shown at the bottom of Fig. 1.) Two examples of NO_2_ concentrations along the Wilton Road transect between Craigmiller Park Road and Mayfield Road for measurement periods with contrasting average wind speeds are shown in Fig. 2. Concentrations of NO_2_ are greater at 0.8 m than at 2.0 m for the sites closest to Craigmiller Park Road (0.4 m away) and to Mayfield Road (0.5 m away), but a significant vertical concentration difference does not persist at the next closest sites to the main roads (4.7 m from Craigmiller Park Road and 11.5 m from Mayfield Road).

A notable feature of Fig. 2 is the different horizontal fall-offs in NO_2_ concentration from the main roads. During two of the five sets of measurements along Wilton Road, background concentrations were reached between 100 and 150 m from Craigmiller Park Road, as exemplified by the measurements in Fig. 2a. Average wind speeds during these two periods were 0.76 and 1.26 m s^−1^. For the other three sets of measurements, during which average wind speeds were 2.25, 2.83, and 3.27 m s^−1^, background concentrations were reached in shorter distances, as exemplified by the data in Fig. 2b.

**Fig. 3:**
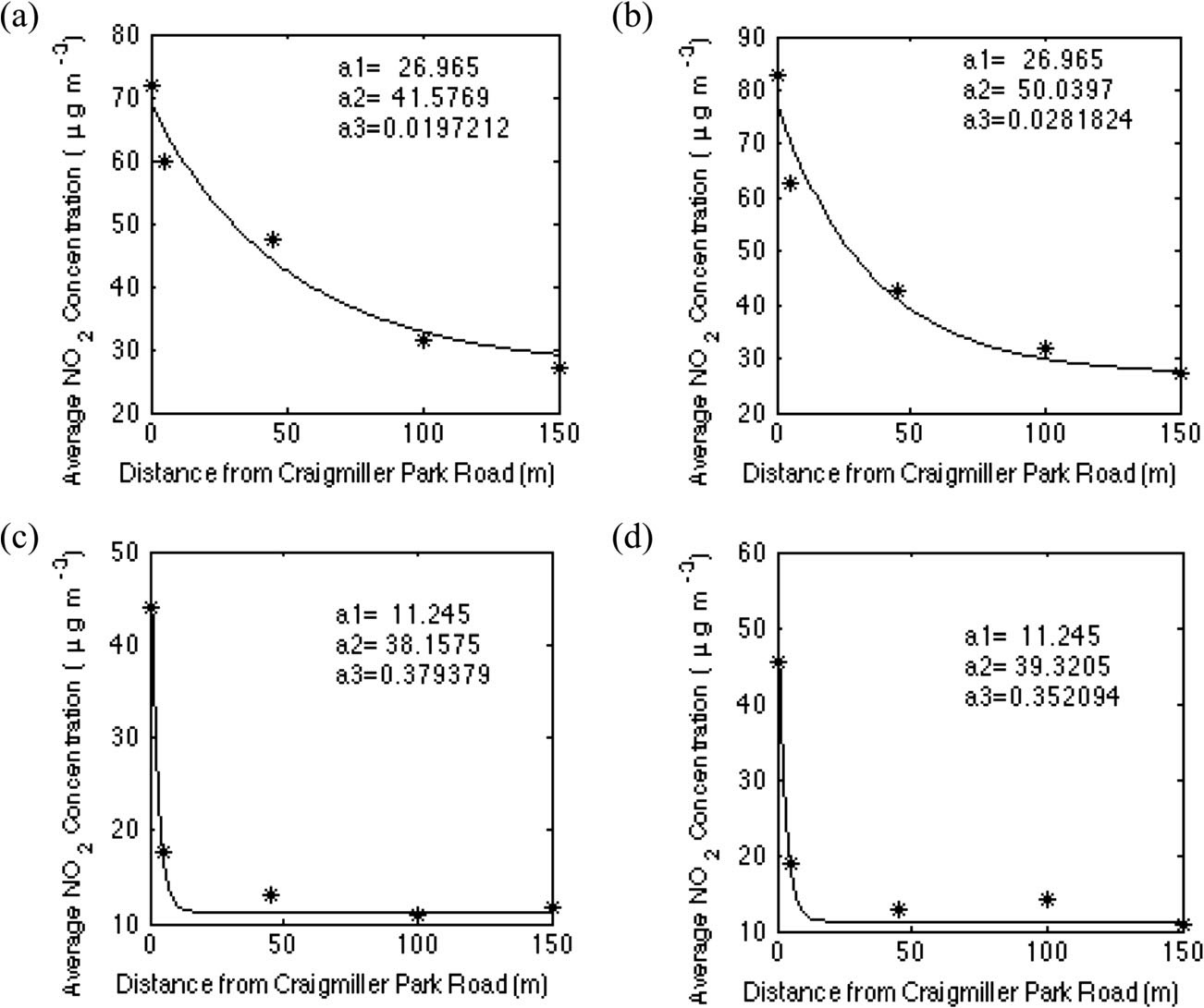
Examples of the fit of the three-parameter exponential given in the text to the fall-off in NO_2_ concentration along the first 150 m of Wilton Road away from Craigmiller Park Road. **a**, **b** Fit to the measurements in the week with the lowest average wind speed (0.76 m s^−1^); **c**, **d** fit to the measurements in the week with the highest average wind speed (3.27 m s^−1^). **a**, **c** Measurements at 2.0 m above the ground; **b**, **d** measurements at 0.8 m above the ground

In general terms, these NO_2_ concentration horizontal fall-off distances of ∼100 m from major roads are similar to those reported previously (Bell and Ashenden 1997; Glasius et al. 1999). However, further analysis shows that the variation in fall-off differences in the current work is associated with variation in the average wind speed. An exponential equation of the form

$$ y={a}_1+{a}_2 \exp \left(-{a}_3x\right) $$

was fitted to the NO_2_ concentrations up to 150 m along Wilton Road for both measurement heights for each exposure, where *y* is the NO_2_ concentration (μg m^−3^) and *x* the distance from Craigmiller Park Road (m). Parameter *a*_1_, which represents the background concentration (μg m^−3^), was fixed in the fittings to the average NO_2_ concentration at the two heights at 150 m along Wilton Road. This ensured that the fittings to concentrations at 2.0 and 0.8 m above the ground converged to a common background concentration, consistent with the observation of no significant vertical concentration gradient at large distances from the roadside. Parameter *a*_2_ represents the additional concentration contribution from traffic at *x* = 0 (μg m^−3^), and *a*_3_ is the horizontal decay constant (m^−1^). Figure 3 illustrates example results for the fittings for the measurement periods with the lowest and highest wind speeds. Using the fitted parameters generated, the NO_2_ concentrations at the roadside (*x* = 0 m) were calculated and compared to the background concentrations *a*_1_. On average, roadside concentrations at 2.0 m from the ground were 3.3 times higher than background concentrations, and concentrations at 0.8 m from the ground were 3.7 times higher than background. These findings are similar to those of Glasius et al. (1999) who reported that NO_2_ concentrations near busy roads exceeded background concentrations by 2.9 times.

**Fig. 4:**
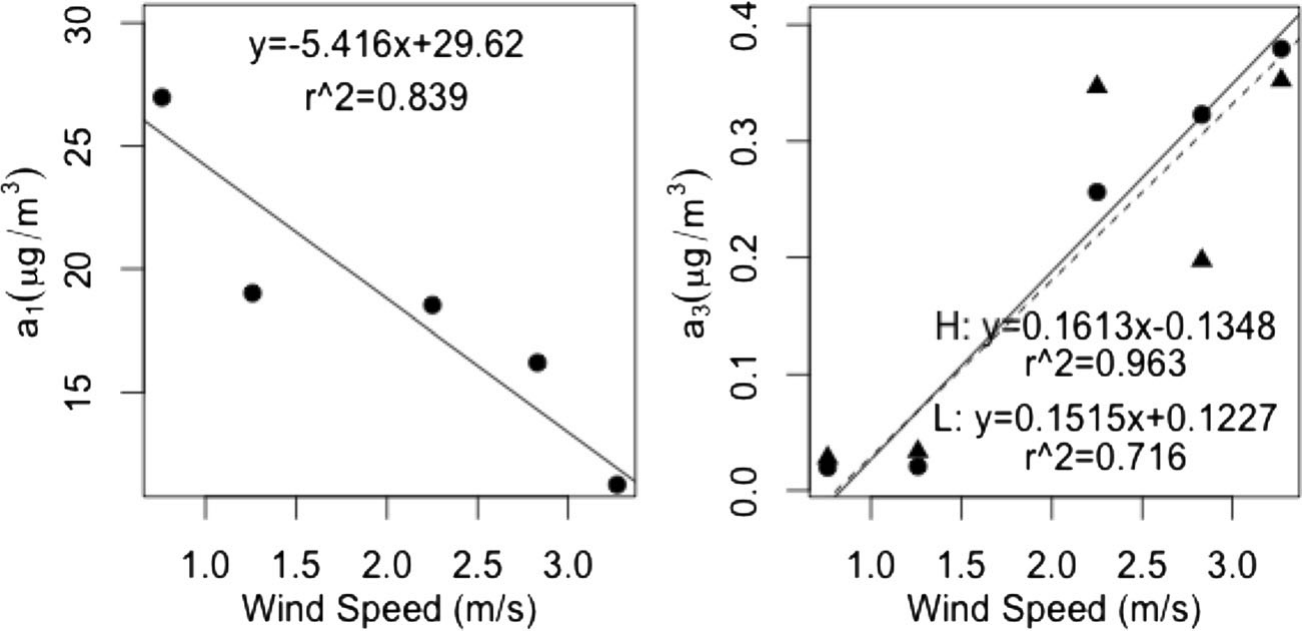
Standard major axis linear regressions of the fitted equation parameters *a*_1_ and *a*_3_ on average wind speed during the NO_2_ measurement. The same value of *a*_1_ is used in fittings to NO_2_ data at each height (see text). For the plot of *a*_3_ values, *circular points* and *solid lines* are for NO_2_ concentrations at 2.0 m above the ground (high, *H*), while *triangular points* and *dashed lines* are for NO_2_ concentrations at 0.8 m above the ground (low, *L*)

Scatter plots comparing wind speed with *a*_1_, the fitted background concentrations, and with *a*_3_, the fitted horizontal decay constants, are shown in Fig. 4. The background concentrations, which varied between 11 and 27 μg m^−3^, were strongly anti-correlated with average wind speed (*r* = −0.92). The decay constant varied between 0.02 and 0.4 m^−1^ at 2.0 m above the ground and between 0.03 and 0.4 m^−1^ at 0.8 m above the ground, and was also strongly correlated with wind speed (*r* = 0.98 and 0.85 for transects at 2.0 and 0.8 m above the ground). In contrast, the values for the fitted parameter, *a*_2_, the contribution from local traffic, were not correlated with average wind speed. These observations show that greater wind speeds increase the rate and extent of dilution of road traffic NO_2_ emissions. The Old Dalkeith Road transects show that the wind-speed-associated dispersion also affects the near-ground vertical gradient in NO_2_ which persists slightly further with distance from the kerbside at lower wind speed. It is noted that both the horizontal decay constant for NO_2_ concentration away from the main road and the background NO_2_ concentration may depend on wind direction as well as wind speed. However, the temporal pattern (i.e. trend in weekly average) in background NO_2_ concentrations was similar to that at the background network site in Edinburgh. This observation is consistent with the background concentrations being predominantly driven by the prevailing meteorology, i.e. for a weekly average measurement, driven principally by the wind speed during the averaging period. While it cannot be excluded that wind direction also had an effect on the profiles of the NO_2_ concentration transects, first, wind in Edinburgh predominantly derives in the south to west quadrant, and, secondly, the correlations of horizontal decay and background concentrations with wind speed shown in Fig. 4 indicate that wind speed is an important factor.

## Conclusions

Measurements by passive diffusion tubes at differing distances from main roads in Edinburgh, UK, have shown NO_2_ concentrations at 0.8 m above the ground to be significantly greater than NO_2_ concentration at 2.0 m above the ground, for distances up to at least 1.2 m from the kerbside of heavily trafficked (annual average daily traffic counts >12,000) urban roads. Concentrations at the lower height were 5–15 % or a few micrograms per cubic metre greater, on average at the lower height than at the higher height. This has potential health implications for differences in NO_2_ exposure close to heavily trafficked roadsides for adults and children and is the first time, as far as we are aware, that such a difference has been demonstrated. The vertical NO_2_ concentration difference did not persist for distances 2.5 m or greater from the kerbside. Fitting to horizontal transects of NO_2_ concentrations away from main roads demonstrated the strong influence of wind speed in yielding faster fall-off in NO_2_ concentration, and in near-ground vertical gradient in NO_2_, from roadside and lower background NO_2_ concentrations.
